# Combination of KIR2DS4 and *FcγRIIa* polymorphisms predicts the response to cetuximab in *KRAS* mutant metastatic colorectal cancer

**DOI:** 10.1038/s41598-019-39291-2

**Published:** 2019-02-22

**Authors:** A. Borrero-Palacios, A. Cebrián, M. T. Gómez del Pulgar, R. García-Carbonero, P. Garcia-Alfonso, E. Aranda, E. Elez, R. López-López, A. Cervantes, M. Valladares, C. Nadal, J. M. Viéitez, C. Guillén-Ponce, J. Rodríguez, I. Hernández, J. L. García, R. Vega-Bravo, A. Puime-Otin, J. Martínez-Useros, L. Del Puerto-Nevado, R. Rincón, M. Rodríguez-Remírez, F. Rojo, J. García-Foncillas

**Affiliations:** 1 Translational Oncology Division, Oncohealth Institute, Hospital Universitario “Fundación Jimenez Diaz”, Madrid, Spain; 2 Medical Oncology Department, Hospital Virgen del Rocío, Sevilla, Spain; 3 Medical Oncology Department, Hospital Gral. Univ. Gregorio Marañón, Madrid, Spain; 4 Medical Oncology Department, Hospital Universitario Reina Sofía, Córdoba, Spain; 5 Medical Oncology Department, Hospital Vall d’Hebrón, Barcelona, Spain; 6 Medical Oncology Department, Complexo Hospitalario Universitario Santiago de Compostela, Galicia, Spain; 7 Medical Oncology Department, Hospital Clínico Universitario de Valencia, Valencia, Spain; 8 Medical Oncology Department, Complejo Hospitalario Universitario A Coruña, Galicia, Spain; 9 Medical Oncology Department, Hospital Clínic i Provincial de Barcelona, Barcelona, Spain; 10 Medical Oncology Department, Hospital Universitario Central de Asturias, Asturias, Spain; 11 Medical Oncology Department, Hospital Universitario Ramón y Cajal, Madrid, Spain; 12 Medical Oncology Department, Clínica Universitaria de Navarra, Navarra, Spain; 13 Medical Oncology Department, Complejo Hospitalario de Navarra, Navarra, Spain; 14 Oncology, Medical Unit, Merck S.L, an affiliate of Merck KGaA, Darmstadt, Germany; 15 Anatomopathology Department, Hospital Universitario “Fundación Jimenez Diaz”, Madrid, Spain

**Keywords:** Predictive markers, Colorectal cancer

## Abstract

Cetuximab is a standard-of-care treatment for *RAS* wild-type metastatic colorectal cancer (mCRC) but not for those harbor a *KRAS* mutation since MAPK pathway is constitutively activated. Nevertheless, cetuximab also exerts its effect by its immunomodulatory activity despite the presence of RAS mutation. The aim of this study was to determine the impact of polymorphism *FcγRIIIa* V158F and killer immunoglobulin-like receptor (KIR) genes on the outcome of mCRC patients with *KRAS* mutations treated with cetuximab. This multicenter Phase II clinical trial included 70 mCRC patients with *KRAS* mutated. We found *KIR2DS4* gene was significantly associated with OS (HR 2.27; 95% CI, 1.08–4.77; P = 0.03). In non-functional receptor homozygotes the median OS was 2.6 months longer than in carriers of one copy of full receptor. Multivariate analysis confirmed *KIR2DS4* as a favorable prognostic marker for OS (HR 6.71) in mCRC patients with *KRAS* mutation treated with cetuximab. These data support the potential therapeutic of cetuximab in *KRAS* mutated mCRC carrying non-functional receptor *KIR2DS4* since these patients significantly prolong their OS even after heavily treatment. *KIR2DS4* typing could be used as predictive marker for identifying RAS mutated patients that could benefit from combination approaches of anti-EGFR monoclonal antibodies and other immunotherapies to overcome the resistance mediated by mutation in RAS.

## Introduction

Colorectal cancer (CRC) is the third most common cancer worldwide and the second most common in Europe with 447,136 new cases in 2012^1^. Specifically in Spain the incidence was 32,240 new cases. If trends continue by December 31, 2020, Spain will have approximately 37,229 new CRC cases^2^. Approximately 20% of patients present metastatic disease (mCRC) at diagnosis^3^.

Deregulation of the mitogen-activated protein kinase pathway is required for the development of CRC and it is activated by binding of growth factor ligands to the epidermal growth factor receptor (EGFR)^4^. Cetuximab is a monoclonal antibody that binds to the extracellular domain of EGFR, causing inhibition of the downstream signaling pathway, and is a widely used biological treatment for mCRC^5^. Mutations in *KRAS* result in constitutive activation of the pathway, since it is expected that patients with mutations in *KRAS* will not benefit from cetuximab treatment^6^. Despite this rationale, approximately 30% of mCRC patients with mutations in *KRAS* do benefit from cetuximab^7^. Understanding the mechanisms underlying the response of this subgroup to cetuximab could benefit these hard-to-treat patients.

Because cetuximab is an IgG1 monoclonal antibody it also exerts its effect by its immunomodulatory activity in part via antibody-dependent cell-mediated cytotoxicity (ADCC)^8–10^. Cetuximab stimulates ADCC activity when its constant region (Fc) binds to a Natural Killer (NK) cell receptor (CD16/*FcγRIIIa*) leading their own lytic activity on tumor cells^9,11^. Also important downstream immunological responses are possible when CD32A/ *FcγRIIa* receptors are present on dendritic cells and neutrophils leading to priming of tumor antigen-specific cellular immunity^12^. Previous reports suggested that specific polymorphic variants of *FcγR* are associated with the clinical outcome of cancer patients^13,14^. In particular, the *FcγRIIa* H131 allele has been shown to increase the ability of cetuximab to control disease in patients with mutated *KRAS*^8^. The role of *FcγRIIIa* in the ADCC induction by cetuximab in colorectal cancer patients is controversial. While in one study no statistically significant associations between the presence of *FcγRIIIa* and response, progression-free survival (PFS) or *RAS* status in mCRC patients were found^15^, in a subsequent study a tendency for a higher disease control rate (DCR) was shown in patients with the *FcγRIIIa* V-containing genotype^8^. Recently, a different study demonstrated that the *FcγRIIIa* V158V genotype was correlated with a higher ADCC^16^, although sample size was too small to confirm the impact of this association.

A clinical trial with *KRAS* wt mCRC patients treated with cetuximab + Irinotecan showed that ADCC response was not affected by *BRAF* or *NRAS* mutations^17^. Another phase III clinical trial with very similar patients demonstrated that ADCC response scores were higher in patients carrying *FcγRII*a 131H allele *vs*. 131R/R^18^. Furthermore, *FcγRIIIa* 158V carriers were associated with higher cetuximab-mediated ADCC compared to 158F/F. Objective response was significantly higher in both patients carrying the *FcγRIIa* 131H allele and those carrying the *FcγRIIIa* 158V allele. However, survival analysis only revealed longer progression-free survival in patients carrying *FcγRIIIa* 158V allele^18^.

Besides *FcγRIIIa*, Killer immunoglobulin-like receptors (KIRs) are also essential in the immune response against tumor cells^17,18^. They are located on the surface of NK cells and regulate their killing function in the ADCC response^19^. To date, 14 functional KIR genes and two pseudogenes located in the leukocyte receptor complex on chromosome 19q13.4 have been identified. They present a very high degree of structural homology and can be classified as inhibitors or activators of the immune response depending on their cytoplasmic tails. Products of inhibitory KIR genes are characterized by long cytoplasmic tails (“L” KIR genes) and transmit inhibitory signals leading to the general shutdown of NK cell effector functions. Opposite, activating KIR proteins have short cytoplasmic tails (“S” KIR genes) and their signal promotes NK cell activity^20^. The immune-modulating effects depend on the balance of the number and type of receptors exposed on the cell surface^21^. KIR genes are organized in a highly polymorphic multigene family and their combination define two main groups of haplotypes. A haplotypes are characterized by a predominance of genes encoding inhibitory receptors. B haplotypes are more heterogeneous and generally contain several activating genes^22^. A recent study provides evidence of the role of specific variants of KIRs on the response to anti-EGFR treatment in solid tumors including RAS wild type advanced colorectal cancer. Due to the strong immunomodulatory activity of cetuximab we hypothesized that both KIR and *FcγRIIIa* receptors might influence the response of mCRC patients to cetuximab independently of RAS mutation.

This prospective multicenter clinical study aimed to determine whether polymorphisms in CD16/*FcγRIIIa* and/or *KIR* genes could enhance the clinical impact of the *FcγRIIa* polymorphism in mCRC patients with *KRAS* mutated who are treated with cetuximab.

## Results

### Patient characteristics and clinical outcome

Table 1 summarizes the baseline demographic and disease characteristics of the participants. Baseline blood levels were: median 46.50 ng/μL (0–2930) for CEA, median 330 UI/L (150–5882) for LDH and 2.10 mg/L (1–4) for β2-microglobulin. The median time of follow-up was 6.4 months (range: 3.8–10.2 months).

**Table 1:** Baseline characteristics of patients.

	Patients (*N* = 70)
**Age at diagnosis (years)**	
Median (range)	64 (42–82)
**Gender** ***N*** **(%)**	
Male	36 (51.4)
Female	34 (48.6)
**Primary site**	
Colon	52 (74.3)
Rectum	18 (25.7)
**Number of metastatic sites**	
1	16 (22.8)
2	27 (38.6)
3 or more	27 (38.6)
**ECOG performance status**	
0	13 (18.6)
1	51 (72.9)
2	6 (8.6)
**CEA basal**	
≤ULN	8 (11.4)
>ULN	60 (85.7)
N/A	2 (2.9)
**LDH basal**	
≤ULN	35 (50.0)
>ULN	30 (42.9)
N/A	5 (7.1)
**β2 microglobulin basal**	
≤ULN	53 (75.7)
>ULN	8 (11.4)
N/A	9 (12.9)

Abbreviations: CEA, carcinoembryonic antigen; ECOG, Eastern Cooperative Oncology Group; LDH, lactate dehydrogenase; N/A, Not available; ULN, Upper limit of the normal range.

Overall, 68 patients (97.1%) experienced disease progression. Fifty-six (80.0%) patients died during the study period.

Median OS was 6.71 months with a 95% CI of (5.4–8.1) and median PFS was 2.53 with a 95% CI of (2.4–2.7).

### Frequency of V158F FcγRIIIa allele and presence of KIR genes

Twenty-eight out of 70 patients (39.4%) were homozygous for *FcγRIIIa*-158F allele, 36 patients (52.1%) were heterozygous (V/F) and 6 (8.5%) were homozygous for *FcγRIIIa*-158V allele (Table 2). The minor allele frequency of this polymorphism was 34% in our series, which was in concordance with the frequency described in the Spanish population^23^.

**Table 2:** Polymorphisms frequencies.

Polymorphic gene		Genotype frequency N (%)	
FcγRIIIa	FF	FV	VV
V158 F	28 (40)	36 (51.4)	6 (8.6)
**Inhibitory KIR genes**	**+**	**−**	
2DL1	64 (91.4)	6 (8.6)	
2DL2	37 (52.9)	33 (47.1)	
2DL3	63 (90.0)	7 (10.0)	
2DL4^a^	70 (100)	—	
2DL5A	28 (40.0)	42 (60.0)	
2DL5B	20 (28.6)	50 (71.4)	
3DL1	64 (91.4)	6 (8.6)	
3DL2	70 (100)	—	
3DL3	70 (100)	—	
**Activating KIR genes**	**+**	**−**	
2DS1	37 (52.9)	33 (47.1)	
2DS2	36 (51.4)	34 (48.6)	
2DS3*	30 (43.5)	39 (56.5)	
2DS4***f***	14 (20.0)	56 (80.0)	
2DS4***d***	60 (85.7)	10 (14.2)	
2DS5	22 (31.4)	48 (68.5)	
2DL4^a^	70 (100)	—	
3DS1	28 (40.0)	42 (60.0)	
**KIR pseudogenes**	**+**	**−**	
2DP1	64 (91.4)	6 (8.6)	
3DP1***f***	20 (28.6)	50 (71.4)	
3DP1***d***	50 (71.4)	20 (28.6)	

^*^One patient was undetermined; ^a^It has both properties.

Abbreviations: *d*, deleted variant; *f*, full variant.

With regard to *KIR* genes (Table 2), some of the gene variants were present in more than 90% of the patients (*KIR2DL1*, *KIR2DL3*, *KIR2DL4*, *KIR3DL1*, *KIR3DL2*, *KIR3DL3* and *KIR2DP1*). Owing to this high frequency, these genes were excluded from the survival analysis. The remaining *KIR* genes and pseudogene (*KIR3DP1*) were included in the survival analysis.

### Survival analysis

Regarding clinical and biochemical parameters only the number of metastatic sites was significantly correlated by univariate analysis (Table 3).

**Table 3:** Cox regression univariate analysis for the association between baseline characteristics and patients’ outcome.

	Overall survival		Progression-free survival	
	*P*	HR (95% CI)	*P*	HR (95% CI)
Sex (female *vs*. male)	0.568	1.17 (0.69–1.98)	0.549	1.17 (0.71–1.92)
Age (≥60 *vs*. <60)	0.824	0.94 (0.51–1.70)	0.335	1.30 (0.76–2.22)
Tumor primary site (rectum *vs*. colon)	0.687	1.13 (0.63–2.04)	0.466	1.24 (0.70–2.18)
ECOG (1 & 2 *vs*. 0)	0.384	1.38 (0.67–2.821)	0.630	1.16 (0.63–2.15)
CEA (>ULN *vs*. ≤ULN)	0.376	1.47 (0.63–3.45)	0.288	1.54 (0.70–3.40)
LDH (>ULN *vs*. ≤ULN)	0.465	1.23 (0.71–2.15)	0.520	1.18 (0.71–1.98)
β2 microglobulin (>ULN *vs*. ≤ULN)	0.678	0.85 (0.40–1.83)	0.767	0.89 (0.42–1.90)
**Number of metastatic sites**				
2 *vs*. 1	0.069	2.00 (0.95–4.22)	0.172	1.61 (0.81–3.18)
3 or more *vs*. 1	0.009	2.75 (1.28–5.89)	0.007	2.53 (1.29–4.97)

Abbreviations: CEA, carcinoembryonic antigen; CI, confidence interval; ECOG, Eastern Cooperative Oncology Group; HR, Hazard ratio; LDH, lactate dehydrogenase; ULN, Upper limit of the normal range.

**Figure 1:**
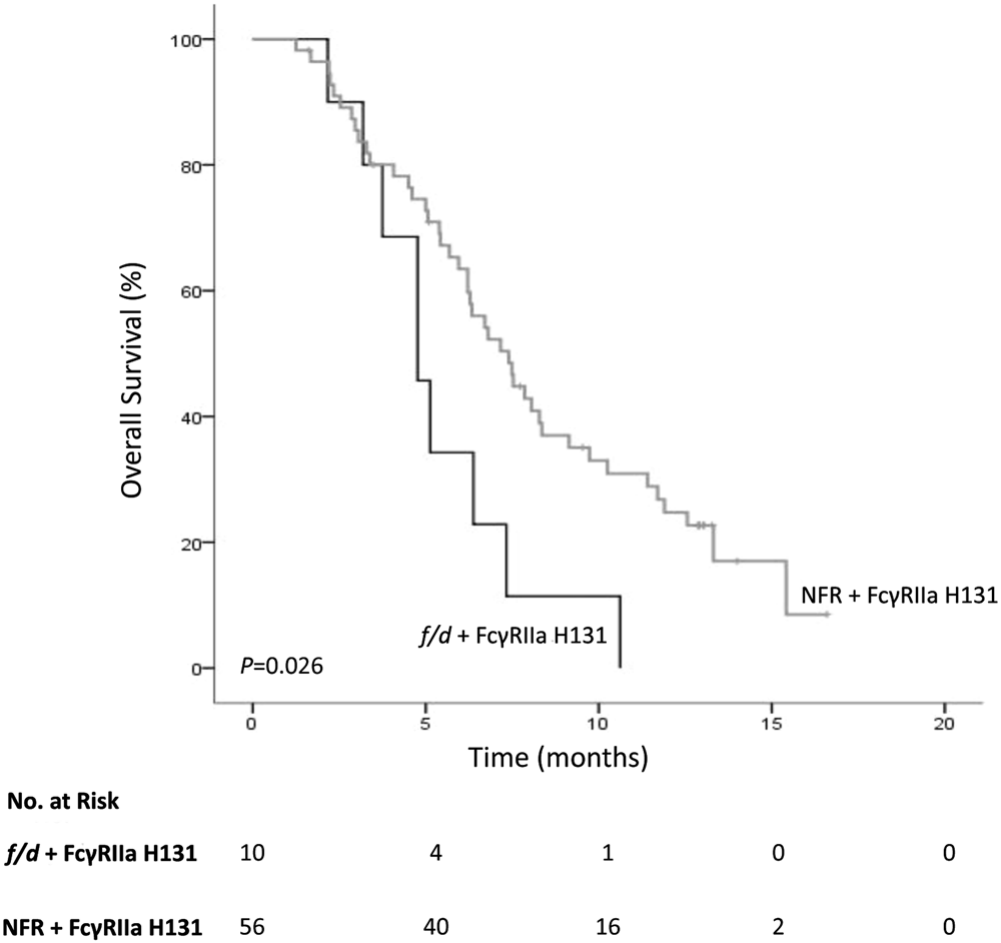
Kaplan–Meier curve for overall survival according to the status of the KIR2DS4 gene. Heterozygous individuals (full variant [*f*] and deleted variant [*d*]; *f*/*d*) were compared with all patients with the non-functional receptor (homozygous deleted variant and without any copy of the gene; NFR). All patients were carriers of *FcγRIIa* H131 polymorphism. The median overall survival among heterozygotes for the KIR2DS4*f* allele was 4.8 months (95% CI, 3.45–6.08) and 7.4 months (95% CI, 5.97–8.82) for patients with NFR (*P* = 0.026; log-rank test).

Cox regression analysis showed that only KIR2DS4 polymorphism was significantly associated with OS (Table 4). *KIR2DS4* encodes two different proteins, a full length variant (*KIR2DS4f*) and a truncated protein with a 22-bp deletion in exon 5 (*KIR2DS4d*) resulting in a protein unable to attach to the cytoplasmic membrane. The genotype distribution in the study population was 50 (71.4%) homozygotes for the *KIR2DS4d*, four (5.7%) homozygotes for the *KIR2DS4f*, 10 (14.28%) heterozygotes (*f/d*) and six (8.6%) patients did not have any copy of the *KIR2DS4* gene. As both homozygotes for the deleted variant and patients with no copy of the gene have a non-functional receptor (NFR), these patients were grouped together. A significantly lower OS was observed for patients with at least one *KIR2DS4f* allele compared with individuals without a functional receptor (median 4.8 months vs. 7.4 months; HR 2.27; 95% CI, 1.08–4.77; *P* = 0.026) (Fig. 1). Since the number of homozygotes for *KIR2DS4f* was very small (n = 4), it was not possible to determine the expected cumulative effect of this genotype.

**Table 4:** Cox regression univariate analysis for the association between polymorphisms in KIR genes or FcγRIIIa and outcome of the patients.

	Overall survival		Time to progression	
	*P*	HR (95% CI)	*P*	HR (95% CI)
FcγRIIIa V158F*	0.864	1.05 (0.61–1.79)	0.363	0.79 (0.48–1.31)
KIR2DL2	0.909	1.03 (0.60–1.76)	0.444	0.83 (0.50–1.35)
KIR2DL5A	0.934	1.02 (0.60–1.76)	0.754	0.92 (0.55–1.54)
KIR2DL5B	0.880	0.96 (0.53–1.73)	0.209	0.70 (0.40–1.22)
KIR2DS1	0.776	1.08 (0.63–1.86)	0.888	0.97 (0.59–1.58)
KIR2DS2	0.967	0.99 (0.58–1.69)	0.349	0.79 (0.48–1.29)
KIR2DS3	0.587	1.16 (0.68–1.97)	0.292	0.76 (0.46–1.26)
KIR2DS4^†^	0.030	2.25 (1.08–4.71)	0.496	1.28 (0.63–2.64)
KIR3DS1	0.520	1.19 (0.70–2.05)	1.000	1.00 (0.60–1.67)
KIR3DP1^¥^	0.888	1.04 (0.59–1.85)	0.562	1.18 (0.67–2.07)

^*^Individuals with at least one valine (FV and VV) were compared with homozygous for phenylalanine (FF). ^†^Carriers of one full variant (KIR2DS4f/d) were compared to individuals with no functional receptor (NFR). ^¥^Individual carrying at least one full variant.

Abbreviations: CI, confidence interval; HR, Hazard ratio.

Multivariate logistic regression model, adjusted for baseline characteristics, showed that 3 or more metastatic sites and KIR2DS4 polymorphism were independent predictors of overall survival (OS) in KRAS mutated mCRC patients carrying FcγRIIa H131 allele and receiving cetuximab (HR 3.03, P = 0.007 and HR 2.17, P = 0.045, respectively) (Table 5).

**Table 5:** Cox regression multivariate analysis for overall survival.

	Overall survival	
	*P*	HR (95% CI)
**Number of metastatic sites**		
2 *vs*. 1	0.085	2.02 (0.91–4.49)
3 or more *vs*. 1	0.007	3.03 (1.36–6.76)
KIR2DS4^†^	0.045	2.17 (1.02–4.63)

^†^Carriers of one full variant (KIR2DS4f/d) were compared to individuals with no functional receptor (NFR).

Abbreviations: CI, confidence interval; *d*, deleted variant; *f*, full variant; HR, Hazard ratio; NFR, non-functional receptor.

Logistic regression and disease control rate (DCR) analysis were also performed; however, no significant result was obtained (data not shown).

## Discussion

Our results support with new evidences the immunomodulatory activity of cetuximab in mCRC patients regardless *KRAS* status. In particular, subjects carrying the non-functional receptor KIR2DS4 showed longer OS than carriers of the full-length variant.

We have previously described higher disease control rate (DCR) in KRAS mutated patients carrying *FcγRIIa* H131 allele; however, only a tendency was observed for patients with the *FcγRIIIa* V-containing genotype. Although this polymorphism is the best-studied biomarker for ADCC its clinical value is not fully confirmed. An analysis in head and neck squamous cell carcinoma, no predictive value for *FcγRIIIa* F158V polymorphism was detected for cetuximab efficacy^24^; and a study in refractory mCRC patients treated with anti-EGFR antibodies did not found significant associations between *FcγRIIIa* polymorphism and clinical outcome^15^. However, two different studies in mCRC patients with KRAS wild-type found a significant difference in outcomes among patients carrying different genotypes of this polymorphism^18,25^. We have evaluated this polymorphism in our cohort of KRAS mutated mCRC patients treated with cetuximab, similarly to Paez *et al*.^15^ we have observed no effect of *FcγRIIIa* V158F on OS or PFS.

Despite the central role of NK-cells in ADCC and the essential role of KIR receptors to modulate NK cell function, the impact of the KIRs on the mCRC clinical outcome have been essentially unexplored. A recent study demonstrated the use of genotyping KIR to predict overall survival to treatment with FOLFIRI in mCRC patients. The authors showed the absence of KIR2DS4 and 3DL1 increased complete response^26^. Apart from this study, there are few more analyses assessing the clinical impact of KIRs in cancer. Siebert *et al*.^27^ found higher level of ADCC and superior event-free survival in Neuroblastoma patients with haplotype B (combination of KIR genes including activating receptor genes) compared to inhibitory haplotype A (a fixed set of gene encoding for inhibitory receptors, except 2DS4). Also in Neuroblastoma patients, Forlenza *et al*.^28^ evaluated whether combinations of KIR3DL1 and HLA ligands could influence patient outcome after treatment with anti-GD2 monoclonal antibody. They evaluated a total of 245 patients and found that single KIR3DL1 did not impact survival but after assessing combinations of KIR3DL1 and HLA-B they found that when receptor and ligand did not interact at the cell surface exhibited the greatest OS and PFS. Similar results were observed by Boudreau *et al*.^29^ when investigated the impact of KIR3DL1/HLA-B combinations in 1,328 patients with acute myelogenous leukemia who underwent hematopoietic cell transplantation (HCT). Patients with weak or non-inhibiting KIR3DL1/HLA-B partnership experienced higher DFS after HCT. Recently, it has been published other study in patients with chronic myeloid leukemia treated with tyrosine kinase inhibitors (TKIs)^30^. The authors hypothesized that KIR and HLA polymorphisms may influence response to TKIs. They did not find association between single KIR genes or the presence of a single functional combination of an inhibitory KIR-HLA genotype with achievement of complete molecular response. However, specific alleles of KIR2DL4, KIR3DL1 and KIR2DS4 were associated with response. A study in non-small cell lung cancer showed patients with absence of KIR2DS4 had longer OS than patients who were positive^31^. A third study demonstrated that donor’s full-length KIR2DS4 allele is associated with lower OS rates and higher relapse incidence in patients with hematological malignancies^32^. Finally, a very recently study showed a protective impact of deleted KIR2DS4 on genetic predisposition to head and neck squamous cell carcinoma (HNSCC)^33^.

In our study, a significant survival advantage was observed for patients without functional KIR2DS4, suggesting a novel favorable and independent prognostic biomarker for overall survival in mCRC patients KRAS mutated and treated with cetuximab. *KIR2DS4* encodes two different allele variants, full-length (KIR2DS4*f*) or deleted (KIR2DS4*d*). A deleted KIR2DS4 is a truncated protein without transmembrane and cytoplasmic domains compared to full-length KIR2DS4 protein. Therefore, this truncated protein is not anchored to cell membrane becoming a soluble variant^21^. The role of this soluble protein remains unclear but it has been suggested that the presence of this truncated protein could minimize the detrimental effects of the KIR2DS4*f* ^33^. Its function could potentially be a decoy that absorbs the available soluble HLA and thus potentially interferes with NK cell function or may be related to an alternative receptor ligand^34,35^. It is important to highlight that *KIR2DS4* is the only activating gene within the KIR A haplotype. Therefore individuals with haplotype A being homozygous for the deleted KIR2DS4 will carry only inhibiting KIRs which has been associated with a protective role against some types of cancer such as HNSCC^33^. Based on our results and those of the above-mentioned studies, we can assume that the lack of functional KIR2DS4 has a positive impact on clinical outcome of cancer patients treated with anti-IgG1 therapies. In our cohort, this influence is stronger when NFR-KIR2DS4 and *FcγRIIa* H131 allele are combined, this effect was also observed in neuroblastoma patients treated with anti-GD_2_ IgG_1_ Ab^27^.

An important factor to consider was the possibility that patients with the G13D mutation skewed our analysis. However, we found no significant differences in OS (*P* = 0.22) or PFS (*P* = 0.70) in G13D patients compared to the rest of patients. It is in agreement with Segelov *et al*.^36^ who did not find statistically significant improvement in disease control at 6 months in patients with G13D mutation chemotherapy-refractory mCRC treated with cetuximab.

One of the limitations of our study that warrant consideration is that it has been performed in a limited cohort. To address whether the combination of *FcγRIIa* and *KIR2DS4* could be a suitable predictive marker for *KRAS* mutated patients, it should be confirmed in large-scale prospective studies.

In conclusion, the use of *FcγRIIa* H131R and KIR2DS4 to identify the subset of *KRAS* mutated patients who might benefit from cetuximab therapy could improve the current management strategies available for these patients. Moreover, these results could explain the observed variability in efficacy of cetuximab in *KRAS* mutated mCRC patients and confirm the important role of ADCC-mediated toxicity to tumor cells by cetuximab.

## Methods

### Patients and trial design

From September 2011 through December 2013, 70 mCRC patients with KRAS mutations were prospectively enrolled in this multicenter Phase II clinical trial (registration number: NCT01450319, 07/10/2011; EudraCT Number: 2010-023580-18). Sample size was calculated using a one-sided test with a significance level of α = 0.05, assuming a recruitment period of 14 months and 12 months of follow-up and considering a minimum frequency of 30% for the KIR and FcγRIIIa-F158V polymorphisms. In order to detect an improvement of overall survival compared to historical controls with at least 80% power, it was estimated a sample size of 70 patients assuming an exponential distribution for OS, a one-sided null hypothesis H0: Median OS <= 5 months and, an actual median OS of 7 months. Primary endpoint was OS, and the secondary endpoint was progression-free survival.

This study was approved by the Institutional Ethical Committee at the University Hospital Fundacion Jimenez Diaz (authorization number EC 02-12 IIS-FJD) and accepted by the other 12 participating hospitals located across Spain. All of them were general hospitals and publically funded excepting “Clínica Universitaria of Navarra” that was private. Written informed consent was obtained from all patients before enrollment. All methods were carried out in accordance with the approved guidelines and regulations.

The patients included in this study were eligible taking into account the inclusion criteria: (A) histologically confirmed mCRC with confirmed *KRAS* mutation; (B) positive EGFR expression; (C) carrier of at least one histidine at position 131 in *FcγRIIa*; (D) aged 18 years or older; (E) Eastern Cooperative Oncology Group (ECOG) performance status of 0–2. All patients had adequate bone marrow, renal and liver functions and were refractory to at least two lines of treatment including standard chemotherapy.

Major exclusion criteria were previous anti-EGFR monoclonal antibody-based therapy, presence of brain metastases or evidence of toxicity greater than grade 1 caused by previous treatment.

After enrollment, cetuximab was administered as an intravenous dose of 500 mg/m^2^ of body surface area, every two weeks until unacceptable toxicity, disease progression, death or revocation of the informed consent.

### Baseline measurements

Peripheral blood was collected in 5-ml tubes with EDTA. Blood levels of carcinoembryonic antigen (CEA), lactate dehydrogenase (LDH) and β2-microglobulin levels were evaluated before initiation of cetuximab therapy as they have been described as prognostic factors in colorectal cancer^37–39^. Patients were grouped according to the upper limit of the normal range (ULN) for each measure (>5 mcg/L for CEA, 333 UI/L for LDH and 3 mg/L for β2-microglobulin).

### *FcγRIIIa* V158F polymorphism and KIRs genotyping

Genomic DNA extraction was performed using the QIAamp DNA Blood Mini Kit (Qiagen). The purified DNA was quantified with a NanoDrop 3.0 spectrophotometer (Nucliber).

The *FcγRIIIa* genotype was determined using the TaqMan Allelic Discrimination Assay (assay code C_25815666_10, Applied Biosystems) according to the manufacturer’s instructions. After thermal cycling, allelic determination was performed using the 7500 Fast real-time PCR instrument (Applied Biosystems). Samples of known genotype were included as the positive control.

The 17 *KIR* genes were determined using the *KIR* genotyping SSP Kit (Applied Biosystems) according to the manufacturer’s protocol. After thermal cycling the genotype-specific PCR products were resolved using 2% agarose gels and interpreted according to the manufacturer’s instructions.

### Statistical Methods

Overall survival was defined as the time from enrollment until death from any cause. Progression-free survival was calculated as the time from enrollment until disease progression, or death due to any cause. In the absence of confirmation of disease progression or death, the participant was censored at the last contact, known to be alive and progression free. Those patients who started a new treatment (different from cetuximab) were censored at the date of starting the new treatment.

Univariate analysis was performed to assess the effect of genetic markers and clinical variables on the prediction of outcome. Only variables that were statistically significant by univariate analysis were considered as covariates in the multivariate Cox regression model. The survival probability was estimated using the Kaplan-Meier method, and the log-rank test was used to test the differences between the subgroups.

Data analysis was performed using the SPSS statistics version 20.0 software package.

## References

[1] Ferlay, J. *et al*. GLOBOCAN 2012 v1.0, Cancer Incidence and Mortality Worldwide: IARC CancerBase No. 11. International Agency for Research on Cancer, Lyon (France); 2013 [accessed 2018 January 31]. http://globocan.iarc.fr.

[2] GLOBOCAN 2012: Estimated Cancer Incidence, Mortality and Prevalence Worldwide in 2012. ARCI: OMS; [accessed 2018 January 31] http://globocan.iarc.fr/Default.aspx.

[3] Cartwright TH (2012). Treatment decisions after diagnosis of metastatic colorectal cancer. Clin. Colorectal Cancer..

[4] Attar BM, Atten MJ, Holian O (1996). MAPK activity is down-regulated in human colon adenocarcinoma:correlation with PKC activity. Anticancer Res..

[5] Di Fiore F, Sesboüé R, Michel P, Sabourin JC, Frebourg T (2010). Molecular determinants of anti-EGFR sensitivity and resistance in metastatic colorectal cancer. Br. J. Cancer..

[6] Karapetis CS (2008). K-ras mutations and benefit from cetuximab in ad-vanced colorectal cancer. N. Engl. J. Med..

[7] Qiu LX (2010). Predictive and prognostic value of KRAS mutations in met-astatic colorectal cancer patients treated with cetuximab: a meta-analysis of 22 studies. Eur. J. Cancer..

[8] Rodríguez J (2012). Fc gamma receptor polymorphisms as predictive mark-ers of Cetuximab efficacy in epidermal growth factor receptor downstream-mutated metastatic colorectal cancer. Eur. J. Cancer..

[9] Ferris RL (2017). Rationale for combination of therapeutic antibodies tar-geting tumor cells and immune checkpoint receptors: Harnessing innate and adaptive immunity through IgG1 isotype immune effector stimulation. Cancer Treat. Rev..

[10] Trivedi S (2016). Anti-EGFR Targeted Monoclonal Antibody Isotype Influ-ences Antitumor Cellular Immunity in Head and Neck Cancer Patients. Clin. Cancer Res..

[11] Desjarlais JR, Lazar GA, Zhukovsky EA, Chu SY (2007). Optimizing en-gagement of the immune system by anti-tumor antibodies: an engineer’s perspective. Drug Discov. Today..

[12] Lee SC, Srivastava RM, López-Albaitero A, Ferrone S, Ferris RL (2011). Natural killer (NK):dendritic cell (DC) cross talk induced by therapeutic monoclonal antibody triggers tumor antigen-specific T cell immunity. Im-munol. Res..

[13] Bibeau F (2009). Impact of Fc{gamma}RIIa-Fc{gamma}RIIIa polymor-phisms and KRAS mutations on the clinical outcome of patients with meta-static colorectal cancer treated with cetuximab plus irinotecan. J. Clin. On-col..

[14] Cartron G (2002). Therapeutic activity of humanized anti-CD20 monoclonal antibody and polymorphism in IgG Fc receptor FcgammaRIIIa gene. Blood..

[15] Paez D (2010). Immunoglobulin G fragment C receptor polymorphisms and KRAS mutations: are they useful biomarkers of clinical outcome in ad-vanced colorectal cancer treated with anti-EGFR-based therapy. Cancer Sci..

[16] Negri FV (2014). Role of immunoglobulin G fragment C receptor polymor-phism-mediated antibody-dependent cellular cytotoxicity in colorectal cancer treated with cetuximab therapy. Pharmacogenomics J..

[17] Lo Nigro C (2016). Evaluation of antibody-dependent cell-mediated cytotox-icity activity and cetuximab response in KRAS wild- type metastatic colo-rectal cancer patients. World J. Gastrointest. Oncol..

[18] Trotta AM (2016). Prospective Evaluation of Cetuximab-Mediated Anti-body-Dependent Cell Cytotoxicity in Metastatic Colorectal Cancer Patients Predicts Treatment Efficacy. *Cancer*. Immunol. Res..

[19] Eriksson M (1999). Inhibitory receptors alter natural killer cell interactions with target cells yet allow simultaneous killing of susceptible targets. J. Exp. Med..

[20] Bakker AB, Phillips JH, Figdor CG, Lanier LL (1998). Killer cell inhibitory receptors for MHC class I molecules regulate lysis of melanoma cells medi-ated by NK cells, gamma delta T cells, and antigen-specific CTL. J. Immu-nol..

[21] Shilling HG (2002). Allelic polymorphism synergizes with variable gene con-tent to individualize human KIR genotype. J. Immunol..

[22] Martin AM, Freitas EM, Witt CS, Christiansen FT (2000). The genomic organi-zation and evolution of the natural killer immunoglobulin-like receptor (KIR) gene cluster. Immunogenetics..

[23] Lopez-Escamez JA (2011). Polymorphisms of CD16A and CD32 Fcγ recep-tors and circulating immune complexes in Ménière’s disease: a case-control study. BMC Med. Genet..

[24] Srivastava RM (2013). Cetuximab-activated natural killer and dendritic cells collaborate to trigger tumor antigen-specific T-cell immunity in head and neck cancer patients. Clin. Cancer Res..

[25] Calemma R (2012). Fc gamma receptor IIIa polymorphisms in advanced colorectal cancer patients correlated with response to anti-EGFR antibodies and clinical outcome. J. Transl. Med..

[26] De RV (2014). Genetic diversity of the KIR/HLA system and outcome of patients with metastatic colorectal cancer treated with chemotherapy. PLoS One.

[27] Siebert N (2016). Neuroblastoma patients with high-affinity FCGR2A, -3A and stimulatory KIR 2DS2 treated by long-term infusion of anti-GD(2) an-tibody ch14.18/CHO show higher ADCC levels and improved event-free survival. Oncoimmunology..

[28] Forlenza CJ (2016). KIR3DL1 Allelic Polymorphism and HLA-B Epitopes Modulate Response to Anti-GD2 Monoclonal Antibody in Patients With Neuroblastoma. J. Clin. Oncol..

[29] Boudreau JE (2017). KIR3DL1/HL A-B Subtypes Govern Acute Mye-logenous Leukemia Relapse After Hematopoietic Cell Transplantation. J Clin Oncol..

[30] Ureshino H (2018). Allelic Polymorphisms of KIRs and HLAs Predict Fa-vorable Responses to Tyrosine Kinase Inhibitors in CML. Cancer Immunol. Res..

[31] He Y, Bunn PA, Zhou C, Chan D (2016). KIR 2D (L1, L3, L4, S4) and KIR 3DL1 protein expression in non-small cell lung cancer. Oncotarget..

[32] Burek Kamenaric M (2017). The impact of KIR2DS4 gene on clinical out-come after hematopoietic stem cell transplantation. Hum. Immunol..

[33] Barani S, Khademi B, Ashouri E, Ghaderi A (2018). KIR2DS1, 2DS5, 3DS1 and KIR2DL5 are associated with the risk of head and neck squamous cell carcinoma in Iranians. Hum. Immunol..

[34] Maxwell LD, Wallace A, Middleton D, Curran MD (2002). A common KIR2DS4 deletion variant in the human that predicts a soluble KIR mole-cule analogous to the KIR1D molecule observed in the rhesus monkey. Tis-sue Antigens..

[35] Katz G (2004). MHC class I-independent recognition of NK-activating re-ceptor KIR2DS4. J. Immunol..

[36] Segelov E (2016). ICECREAM:randomised phase II study of cetuximab alone or in combination with irinotecan in patients with metastatic colorectal cancer with either KRAS, NRAS, BRAF and PI3KCA wild type, or G13D mutated tumours. BMC Cancer..

[37] Giessen C (2013). Evaluation of prognostic factors in liver-limited metastatic colorectal cancer: a preplanned analysis of the FIRE-1 trial. Br. J. Cancer..

[38] Eker B (2015). Factors affecting prognosis in metastatic colorectal cancer patients. Asian Pac. J. Cancer Prev..

[39] Shrout J (2008). beta(2)microglobulin mRNA expression levels are prognostic for lymph node metastasis in colorectal cancer patients. Br. J. Cancer..

