# Author Correction: Combination of KIR2DS4 and *FcγRIIa* polymorphisms predicts the response to cetuximab in *KRAS* mutant metastatic colorectal cancer

**DOI:** 10.1038/s41598-019-43809-z

**Published:** 2019-05-17

**Authors:** A. Borrero-Palacios, A. Cebrián, M. T. Gómez del Pulgar, R. García-Carbonero, P. Garcia-Alfonso, E. Aranda, E. Elez, R. López-López, A. Cervantes, M. Valladares, C. Nadal, J. M. Viéitez, C. Guillén-Ponce, J. Rodríguez, I. Hernández, J. L. García, R. Vega-Bravo, A. Puime-Otin, J. Martínez-Useros, L. Del Puerto-Nevado, R. Rincón, M. Rodríguez-Remírez, F. Rojo, J. García-Foncillas

**Affiliations:** 1 Translational Oncology Division, Oncohealth Institute, Hospital Universitario “Fundación Jimenez Diaz”, Madrid, Spain; 2 Medical Oncology Department, Hospital Virgen del Rocío, Sevilla, Spain; 3 Medical Oncology Department, Hospital Gral. Univ. Gregorio Marañón, Madrid, Spain; 4 Medical Oncology Department, Hospital Universitario Reina Sofía, Córdoba, Spain; 5 Medical Oncology Department, Hospital Vall d’Hebrón, Barcelona, Spain; 6 Medical Oncology Department, Complexo Hospitalario Universitario Santiago de Compostela, Galicia, Spain; 7 Medical Oncology Department, Hospital Clínico Universitario de Valencia, Valencia, Spain; 8 Medical Oncology Department, Complejo Hospitalario Universitario A Coruña, Galicia, Spain; 9 Medical Oncology Department, Hospital Clínic i Provincial de Barcelona, Barcelona, Spain; 10 Medical Oncology Department, Hospital Universitario Central de Asturias, Asturias, Spain; 11 Medical Oncology Department, Hospital Universitario Ramón y Cajal, Madrid, Spain; 12 Medical Oncology Department, Clínica Universitaria de Navarra, Navarra, Spain; 13 Medical Oncology Department, Complejo Hospitalario de Navarra, Navarra, Spain; 14 Oncology, Medical Unit, Merck S.L, an affiliate of Merck KGaA, Darmstadt, Germany; 15 Anatomopathology Department, Hospital Universitario “Fundación Jimenez Diaz”, Madrid, Spain

Correction to: *Scientific Reports* 10.1038/s41598-019-39291-2, published online 22 February 2019

The original version of this Article contained an error in the spelling of the author P. Garcia-Alfonso, which was incorrectly given as P. Garcia. This error has now been corrected in the PDF and HTML versions of this Article.

